# Delphinidin-3-*O*-glucoside, an active compound of *Hibiscus sabdariffa* calyces, inhibits oxidative stress and inflammation in rabbits with atherosclerosis

**DOI:** 10.1080/13880209.2021.2017469

**Published:** 2022-02-07

**Authors:** Bo Sun, Fangda Li, Xu Zhang, Wei Wang, Jiang Shao, Yuehong Zheng

**Affiliations:** a Department of Vascular Surgery, Peking Union Medical College Hospital, Chinese Academy of Medical Sciences, Beijing, China; b Department of Vascular Surgery, Weifang People’s Hospital, Weifang, China

**Keywords:** Hypolipidemic effect, antioxidant effect, inflammatory cytokines, high-fat diet

## Abstract

**Context:**

Delphinidin-3-*O*-glucoside (DP) is a bioactive compound of *Hibiscus sabdariffa* L. (Malvaceae) (Roselle) calyces and exerts endothelial protection and lipid-lowering activities, which provided a basis for the prevention and treatment of cardiovascular diseases.

**Objectives:**

To investigate the therapeutic effects of DP against atherosclerosis.

**Materials and methods:**

A rabbit model of atherosclerosis (AS) was established by 12 weeks of a high-fat diet (HFD). The rabbits were divided into five groups: control, AS, simvastatin (4 mg/kg), and two DP groups (10 and 20 mg/kg). After treatment with DP or simvastatin by oral gavage for 12 weeks, the lipid profiles were measured. Histopathological assessment of the aorta was performed by H&E staining. Oxidative stress and inflammation-related markers were analyzed by ELISA kit and real-time RT-PCR.

**Results:**

DP (20 mg/kg) decreased serum TG (2.36 ± 0.66 *vs.* 4.33 ± 0.27 mmol/L for the AS group), TC, LDL-C, and HDL-C (all *p* < 0.05). DP (20 mg/kg) also reduced lipid levels in the liver and aorta. DP (20 mg/kg) down-regulated the mRNA levels of IL-6, VCAM-1, and NF-κB and up-regulated the mRNA levels of GSH-PX and SOD1.

**Conclusions:**

This study proved that DP alleviated the HFD-induced oxidative stress and inflammation in atherosclerosis rabbits. These results provided the scientific basis for developing novel therapies.

## Introduction

Cardiovascular disease affects the quality of life of patients and is a primary cause of mortality and morbidity in recent decades (Evans et al. 2020). Atherosclerosis is the major pathological basis of most cardiovascular diseases, including thromboembolic disease, coronary disease, and ischaemic cardiac-cerebral vascular disease (Hansson 2005). It has been reported that atherosclerosis is induced through the initial accumulation of cholesterol and lipids in the artery wall, and accompanied by atherosclerotic plaques formation (Lusis 2000). A previous report indicated that endothelial dysfunction is the initiating factor involved in the pathogenesis of atherosclerosis (Gimbrone and García-Cardeña 2016). The dysfunctional endothelial cells could induce the secretion of adhesion molecules, which in turn promote the aggregation of inflammatory cells, ultimately aggravating the progression of atherosclerosis (Yamagata 2019). Additional reports showed that vascular endothelial oxidative stress could evoke inflammatory responses and damage endothelial cells, and plays an important role in the pathogenesis of atherosclerosis (Kattoor et al. 2017; Marchio et al. 2019). Therefore, agents or nutraceuticals that improve antioxidant abilities or inhibit the secretion of adhesion molecules and inflammatory factors are potential candidate drugs for the prevention and treatment of atherosclerosis. Lots of drugs are available for the treatment of atherosclerosis. However, long-term use of these drugs may cause side effects (Wiklund et al. 2013). Thus, developing the safety and effectiveness of alternative therapies is urgently needed for the treatment of atherosclerosis.

Preclinical, clinical, and epidemiological research showed that anthocyanins are abundant and widely found in medicinal plants, which possess prevention effects against cardiovascular disease via acting on multiple targets (Lee et al. 2017; Wood et al. 2019), indicating potential protective effects of anthocyanins on atherosclerosis (Garcia and Blesso 2021). *Hibiscus sabdariffa* L. (Malvaceae) (Roselle) is often used in traditional medicine for the treatment of various diseases, such as cancer, hyperlipidaemia, and hypertension (Riaz and Chopra 2018). A previous report indicated that an aqueous extract from *Hibiscus sabdariffa* suppresses the progression of atherosclerosis in rabbits (Chen et al. 2003). Delphinidin-3-*O*-glucoside (DP) is a pharmacologically active compound of *Hibiscus sabdariffa*. It has been reported that DP suppressed oxLDL-induced cell proliferation apoptosis and inhibition in primary human umbilical vein endothelial cells (Jin et al. 2013). DP was previously shown to be a powerful vascular protective agent that could inhibit oxLDL-evoked injury in human umbilical vein endothelial cells (Jin et al. 2014).

Although the anti-atherosclerotic effect of *Hibiscus sabdariffa* was previously studied, the pharmacologically active ingredient responsible for its anti-atherosclerotic effect is still not clear. There is little research on the anti-atherosclerotic effect of DP *in vivo*. Therefore, this research investigates the beneficial effects of oral administration with DP on a rabbit model of atherosclerosis.

## Materials and methods

### Reagents

DP (purity >97%) was purchased from Polyphenols Laboratories (Sandnes, Norway). Simvastatin (Sim) was purchased from Pfizer (NY, USA).

### Experimental animals

Male New Zealand white rabbits (body weight 1.8–2.2 kg; age, three months) were obtained from the Beijing Vital River Laboratory Animal Technology Co., Ltd. (Beijing, China). Animals were housed in the SPF animal house of Peking Union Medical College with a relative humidity of 50–60% and an ambient temperature of 22–26 °C. Tap water and food were provided *ad libitum*. The animal experiment was approved by the Animal Ethics Committee of Peking Union Medical College (Approval number: 20210058).

### Grouping and treatment

After seven days of adaptive feeding, rabbits were randomly assigned into five groups (*n* = 8). Control group (Con): rabbits fed with standard laboratory chow and received physiological saline by oral gavage for 12 weeks. Atherosclerotic model group (AS): AS rabbits received an equal volume of physiological saline by oral gavage for 12 weeks. Low-dose DP treatment group (LDP): AS rabbits received 10 mg/kg/d of DP by oral gavage for 12 weeks. High-dose DP treatment group (HDP): AS rabbits received 20 mg/kg/d of DP by oral gavage for 12 weeks. Sim treatment group (Sim): AS rabbits received 4 mg/kg/d of Sim by oral gavage for 12 weeks. DP and Sim were dissolved in physiological saline and were prepared freshly. The dose of DP was chosen based on a previous study and our preliminary experiment (Jin et al. 2013). AS model group was induced by feeding the rabbits with a high-fat diet (10% egg yolk powder, 10% lard, 3% cholesterol, and 77% standard laboratory chow) for 12 weeks. To promote atherosclerotic plaque formation, those AS rabbits were intravenously injected 200 mg/kg of 10% bovine serum albumin (BSA) four weeks after the experiment began (Rücker et al. 1988). At the end of the 12th week, rabbits were fasted for 12 h, anaesthetized using ketamine hydrochloride (100 mg/kg) and xylazine (10 mg/kg), and then sacrificed. Blood samples were collected from the femoral artery of the rabbits and centrifuged at 3500 × *g* for 5 min. The supernatant was collected to obtain the serum and stored at −80 °C for further analysis. The abdominal aorta, thoracic aorta, and liver tissue were collected and washed with iced physiological saline. A portion of liver and aorta tissue was homogenized for lipid and inflammation-related markers measurement. The remaining aorta tissues were stored at −80 °C for histopathological assessment and RT-PCR.

### Histopathological analysis

The abdominal aorta was fixed in formalin solution (10%) for 24 h. Then, specimens were dehydrated. The paraffin-embedded tissues were sectioned (10 µm thickness) and stained with H&E. Images were captured and obtained using a light microscope.

### Serum biochemical analysis

Serum TG, TC, LDL-C, HDL-C, MDA, CAT, GSH-PX, and SOD were measured using corresponding assay kits according to the manufacturer’s instructions (Nanjing Jiancheng Bio-technology, Nanjing, China). The levels of ICAM-1, MCP-1, VCAM-1, CRP, IL-6, and TNF-α in serum were measured using commercially available ELISA kits following the manufacturer’s instructions (Beijing 4 A Biotech Co., Ltd., Beijing, China).

### Liver and aorta tissue lipid measurement

Liver and aorta tissue (1 g) was added to 10 mL of iced physiological saline for homogenates. The protein levels were measured using a bicinchoninic acid protein assay kit (Nanjing Jiancheng Bio-technology, Nanjing, China). The levels of TG and TC in the tissue homogenates were determined using assay kits according to the manufacturer’s instructions (Nanjing Jiancheng Bio-technology, Nanjing, China).

### Inflammatory markers in aorta

Aorta (1 g) was added to 10 mL of iced physiological saline for homogenates. The levels of ICAM-1, MCP-1, VCAM-1, CRP, IL-6, and TNF-α in the aortic homogenate were determined using commercially available ELISA kits following the manufacturer’s instructions (Beijing 4A Biotech Co., Ltd., Beijing, China).

### Quantitative RT-PCR analysis

The aorta tissue was perfused with PBS and total RNA was extracted using Trizol reagent following the manufacturer’s instructions (Takara, Dalian, China). Total RNA (1 µg) was reverse-transcribed into cDNA using Frist Strand cDNA Synthesis Kit (Qiagen, Valencia, CA, USA) according to the manufacturer’s protocol. Quantitative RT-PCR was performed on an ABI 7300 cycler (Applied Biosystems, CA, USA) using an SYBR Green Master Mix kit (Takara, Dalian, China) according to the manufacturer’s protocol. The sequence of mRNA primer was shown in Table 1. GAPDH was used as the endogenous control.

**Table 1.** Primers used for quantitative real-time PCR.

Gene	Forward primer	Reverse primer
SOD1	AGGTCGGTGTGAACGGATTTG	GGGGTCGTTGATGGCAACA
GSH-Px	CCAGGAGAATGGCAAGAATG	AAGGTAAAGAGCGGGTGAGC
VCAM-1	GAAATGGAATTTGAACCCAAAC	GGCAACATTGACAAAAAGTGG
IL-6	GGTACATCCTCGACGGCATC	GCCTCTTTGCTGCTTTCACAC
NF-κB	TCCGTTACAAGTGCGAGG	TCCCGTGTAGCCATTGAT
GAPDH	GGAGCCAAAAGGGTCATC	CCAGTGAGTTTCCCGTTC

### Statistical analysis

Data were analyzed using the GraphPad Prism 5.0 Software (GraphPad Software, La Jolla, CA, USA) and expressed as mean ± *SD*. Comparisons between groups were performed by the one-way analysis of variance followed by Tukey’s *post-hoc* test. It was considered statistically significant when the *p*-value <0.05.

## Results

### DP improved aortic lesions in HFD-fed rabbits

**Figure 1.**
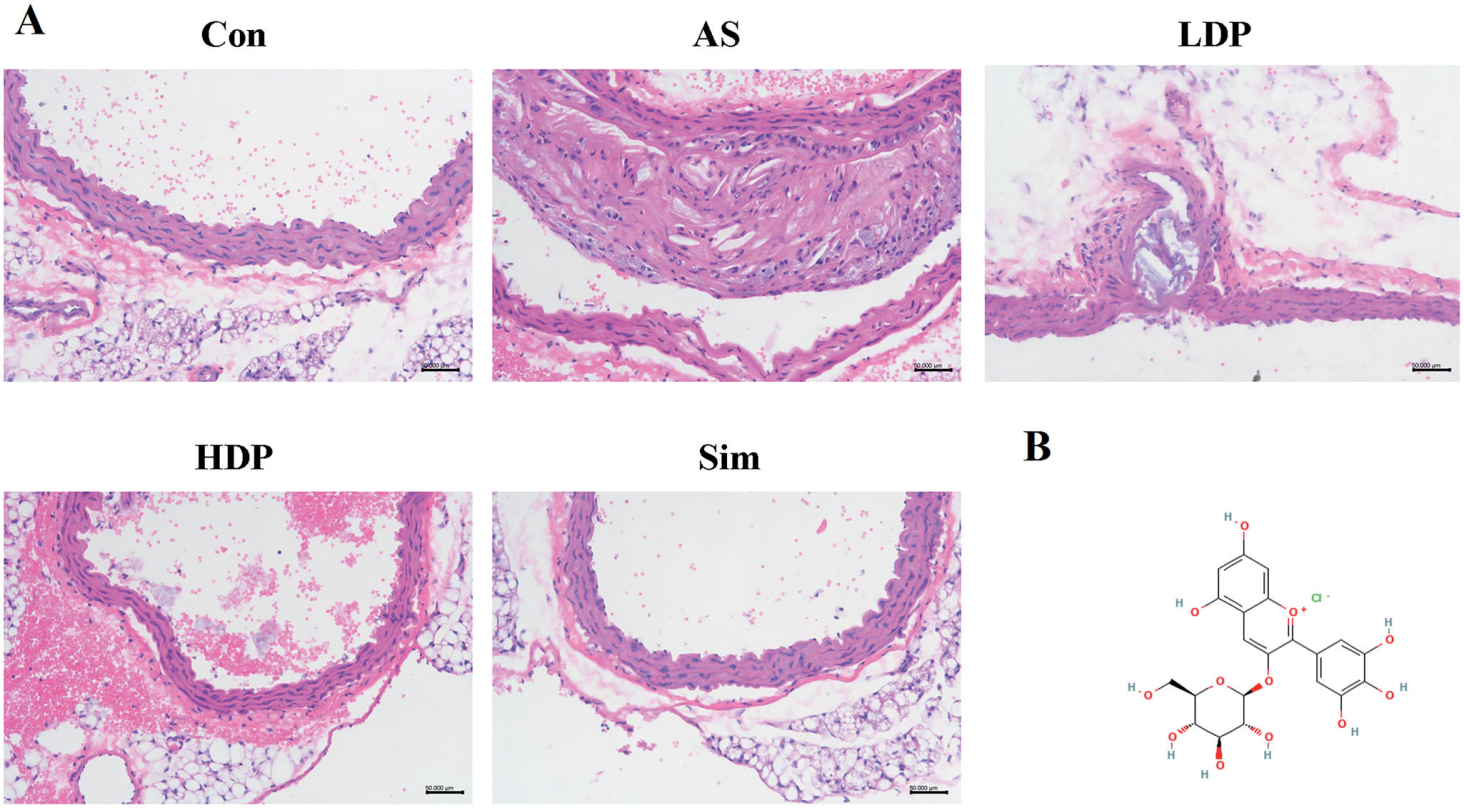
Histological changes of the rabbit abdominal aorta (H&E) after 12 weeks of DP treatment (A), bar = 50 µm, 200× magnification. The molecular structure of delphinidin-3-*O*-glucoside (B).

As shown in Figure 1, the aortic intima of the Con group was morphologically normal and intact, endothelial cells exhibited a regular shape, and no histopathological changes were found. However, the artery tunica media was markedly thickened in the AS group, and disarranged endothelial cells with proliferation were observed in the AS group. Compared to the AS group, the aortic structure of HDP and Sim groups exhibited a relatively intact normal endothelium.

### DP improved serum lipid profile in HFD-fed rabbits

**Figure 2.**
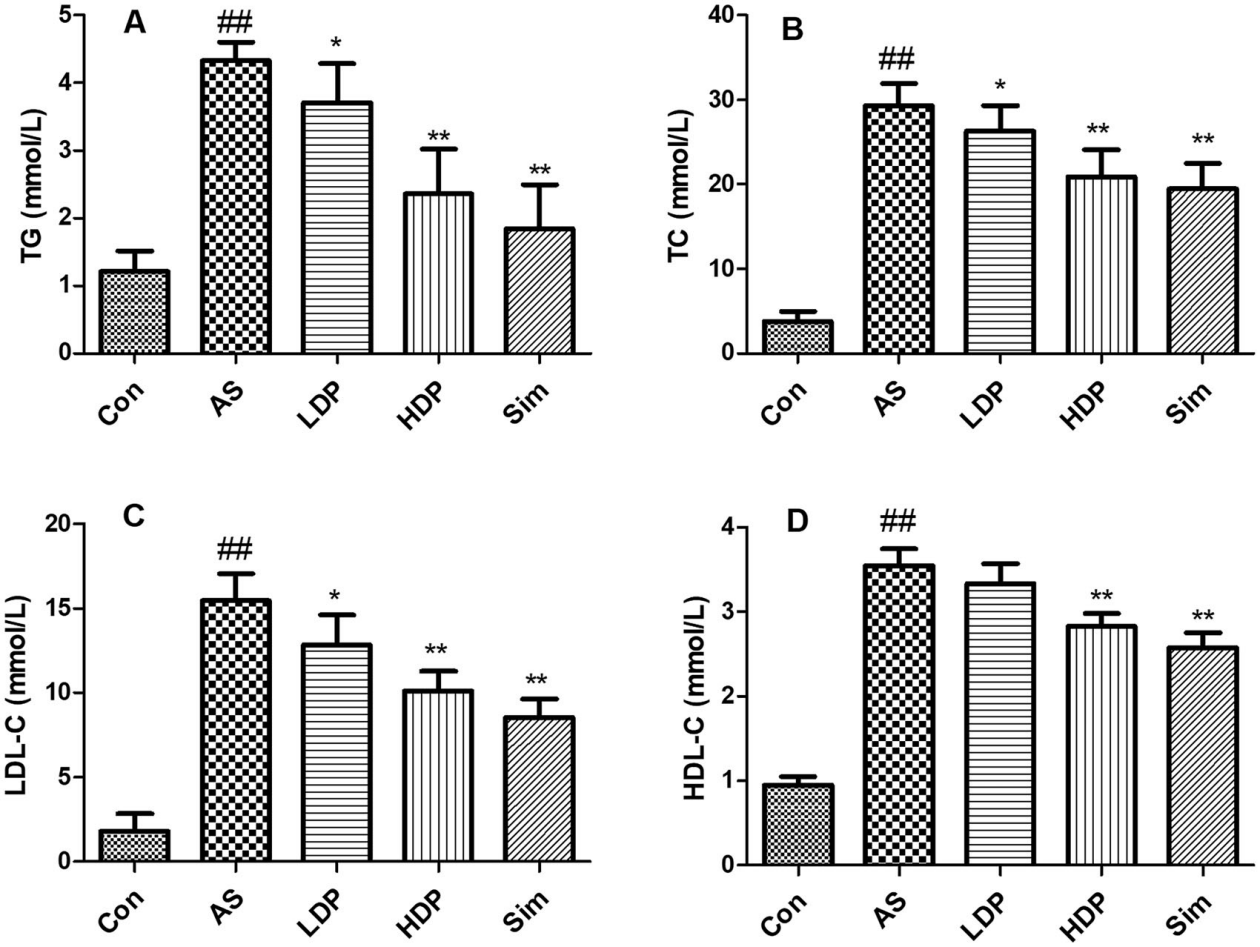
Serum levels of lipid profiles were declined by delphinidin-3-*O*-glucoside (DP). The serum levels of TG (A), TC (B), LDL-C (C), and HDL-C (D) were measured by commercial kits. Values are expressed as mean ± *SD*. ^##^*p* < 0.01 *vs.* control (Con) group; *p < 0.05 *vs.* atherosclerosis (AS) group; **p < 0.01 *vs.* atherosclerosis (AS) group.

As shown in Figure 2, the serum levels of TG, TC, LDL-C, and HDL-C in the AS group increased significantly compared with those in the Con group (*p* < 0.01). Administration of HDP or Sim declined these serum lipid levels compared with those of the AS group (*p* < 0.01).

### DP alleviated liver and aorta lipid profile in as rabbit model

**Figure 3.**
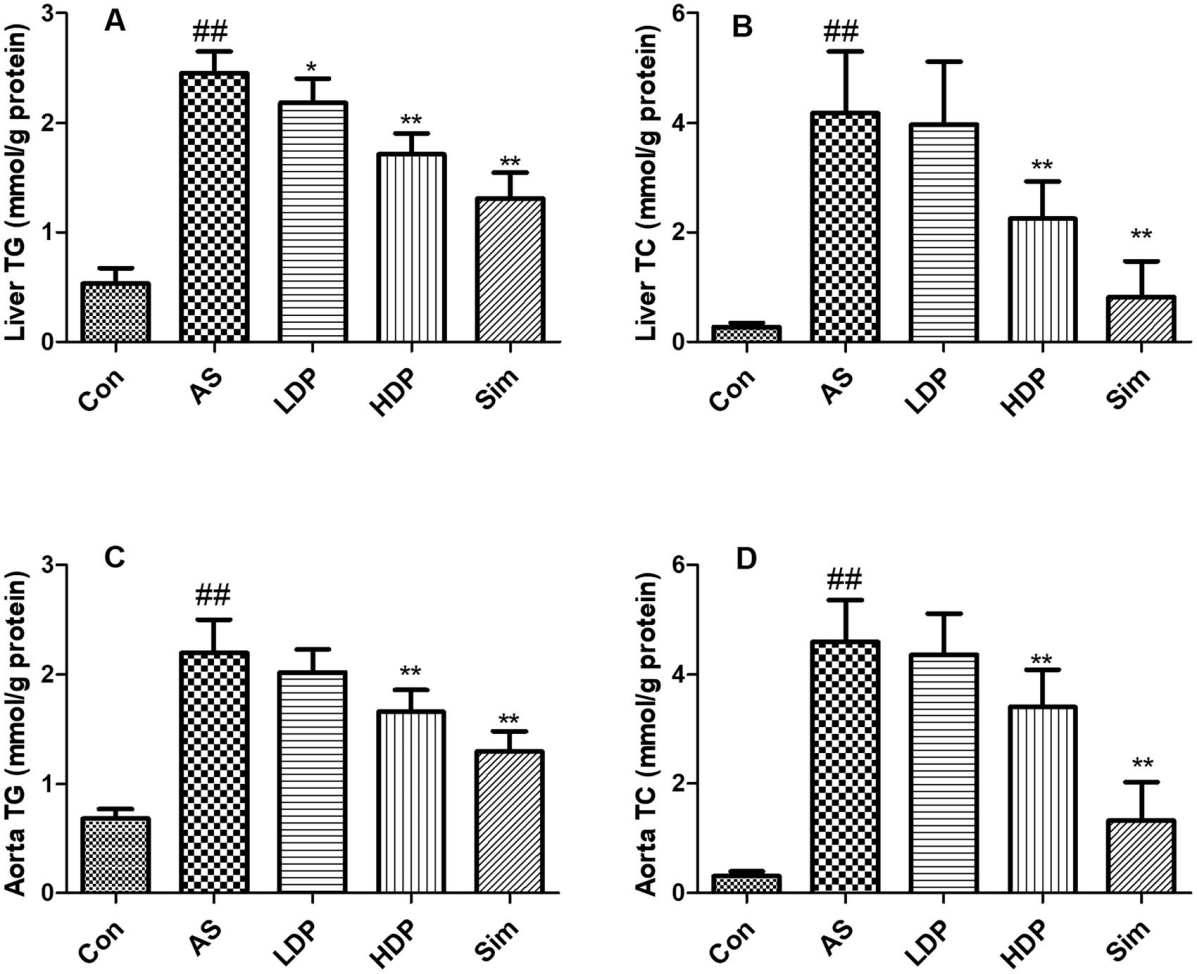
Effects of delphinidin-3-*O*-glucoside (DP) treatment on lipid profiles: liver TG (A), liver TC (B), aorta TG (C), aorta TC (D). Values are expressed as mean ± *SD*. ^##^*p* < 0.01 *vs.* control (Con) group; *p < 0.05 *vs.* atherosclerosis (AS) group; **p < 0.01 *vs.* atherosclerosis (AS) group.

As shown in Figure 3, the hepatic and aortic TG and TC levels in the AS group increased significantly compared with those in the Con group (*p* < 0.01). However, administration of HDP or Sim decreased TG and TC levels compared with those in the AS group (*p* < 0.01).

### DP improved the activities of antioxidative enzyme and decreased MDA content in as rabbit model

**Figure 4.**
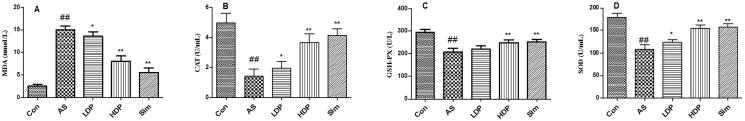
Effects of delphinidin-3-*O*-glucoside (DP) treatment on serum oxidative markers: MDA (A), CAT (B), GSH-Px (C), and SOD (D). Values are expressed as mean ± *SD*. ^##^*p* < 0.01 *vs.* control (Con) group; *p < 0.05 *vs.* atherosclerosis (AS) group; **p < 0.01 *vs.* atherosclerosis (AS) group.

As shown in Figure 4, high oxidative stress was observed in the AS group by the increased MDA content and the inhibited activities of CAT, GSH-PX, and SOD compared to the Con group (*p* < 0.01). However, administration of HDP or Sim improved these antioxidative enzyme activities and reduced MDA levels in HFD-fed rabbits compared to those in the AS group (*p* < 0.01).

### DP inhibited the secretion of inflammatory cytokines in as rabbit model

**Figure 5.**
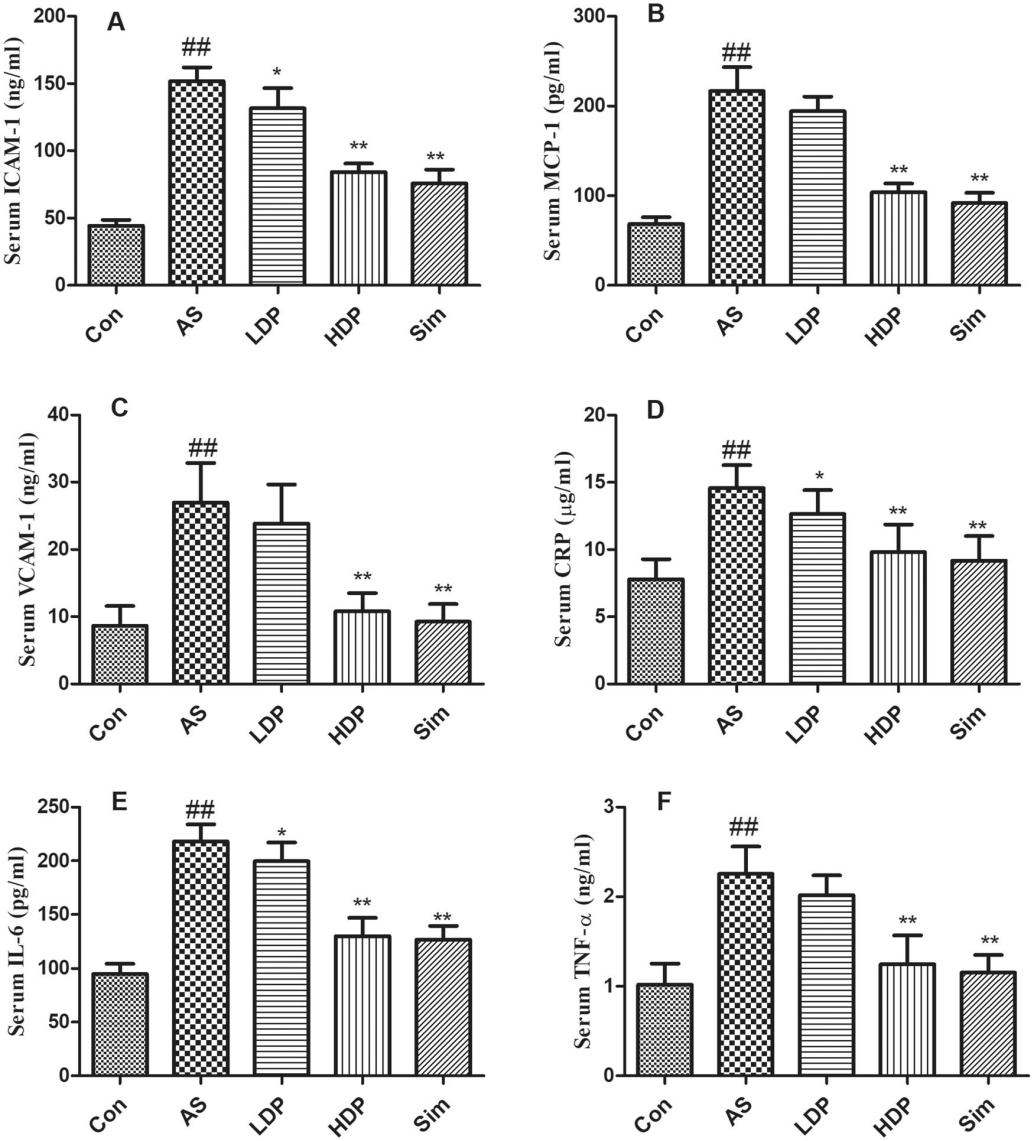
Effects of delphinidin-3-*O*-glucoside (DP) treatment on serum inflammation markers: ICAM-1 (A), MCP-1 (B), VCAM-1 (C), CRP (D), IL-6 (E), and TNF-α (F). Values are expressed as mean ± *SD*. ^##^*p* < 0.01 *vs.* control (Con) group; *p < 0.05 *vs.* atherosclerosis (AS) group; **p < 0.01 *vs.* atherosclerosis (AS) group.

As shown in Figure 5, inflammatory responses were observed in the AS group by the increased serum levels of ICAM-1, MCP-1, VCAM-1, CRP, IL-6, and TNF-α compared to the Con group (*p* < 0.01). However, administration of HDP or Sim inhibited these inflammatory cytokine levels in HFD-fed rabbits (*p* < 0.01).

**Figure 6.**
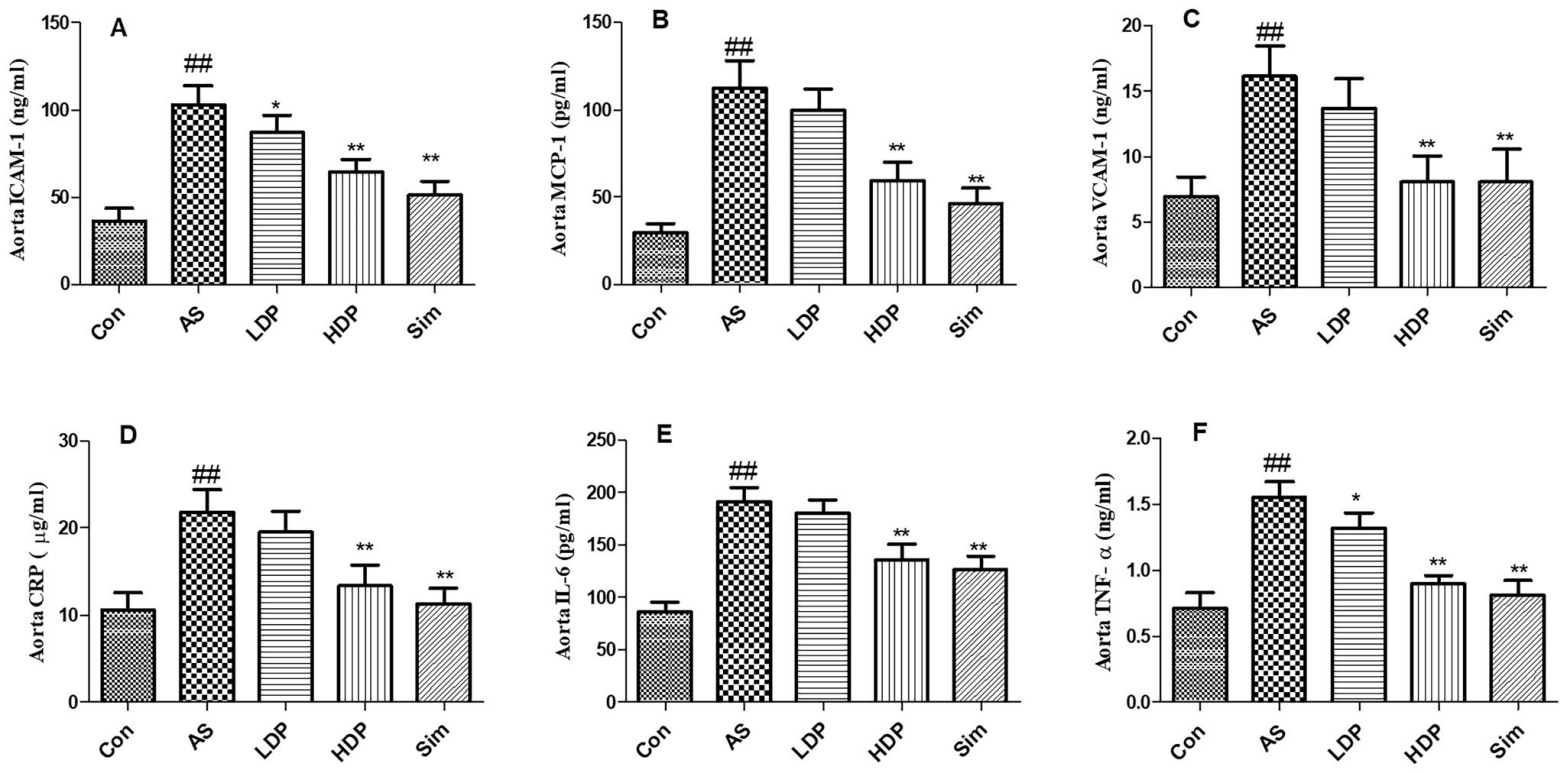
Effects of delphinidin-3-*O*-glucoside (DP) treatment on aorta inflammation markers: ICAM-1 (A), MCP-1 (B), VCAM-1 (C), CRP (D), IL-6 (E), and TNF-α (F). Values are expressed as mean ± *SD*. ^##^*p* < 0.01 *vs.* control (Con) group; *p < 0.05 *vs.* atherosclerosis (AS) group; **p < 0.01 *vs.* atherosclerosis (AS) group.

Furthermore, as shown in Figure 6, inflammation was observed in the AS group by the increased aortic levels of ICAM-1, MCP-1, VCAM-1, CRP, IL-6, and TNF-α compared to the Con group (*p* < 0.01). However, administration of HDP or Sim inhibited these aortic inflammatory cytokine levels in HFD-fed rabbits (*p* < 0.01).

### DP up-regulated the mRNA expression of aortic SOD1 and GSH-Px

**Figure 7.**
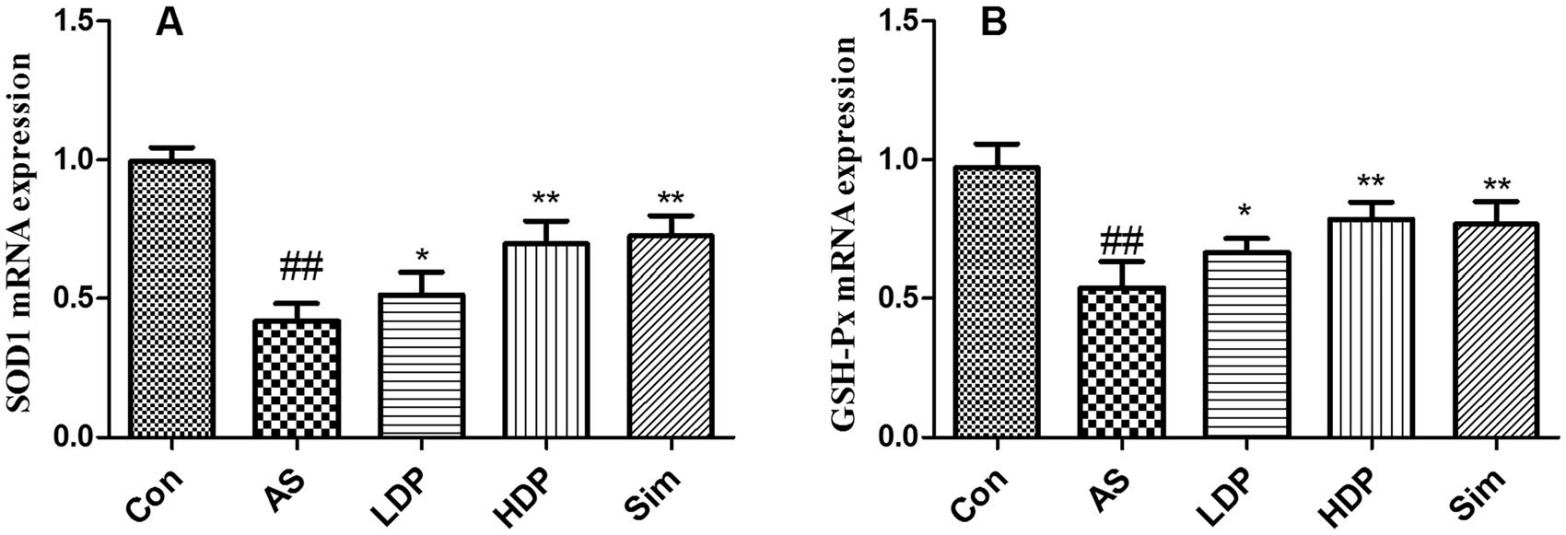
Delphinidin-3-*O*-glucoside (DP) up-regulated the mRNA expression of aortic SOD1 (A) and GSH-Px (B). Values are expressed as mean ± *SD*. ^##^*p* < 0.01 *vs.* control (Con) group; *p < 0.05 *vs.* atherosclerosis (AS) group; **p < 0.01 *vs.* atherosclerosis (AS) group.

As shown in Figure 7, our results indicated that the mRNA expression levels of SOD1 and GSH-Px down-regulated obviously in AS group compared to those in the Con group (*p* < 0.01). However, administration of HDP or Sim up-regulated these aortic antioxidant enzyme-relevant gene expressions in HFD-fed rabbits (*p* < 0.01).

### DP down-regulated the mRNA expression of aortic IL-6, NF-κB, and VCAM-1

**Figure 8.**
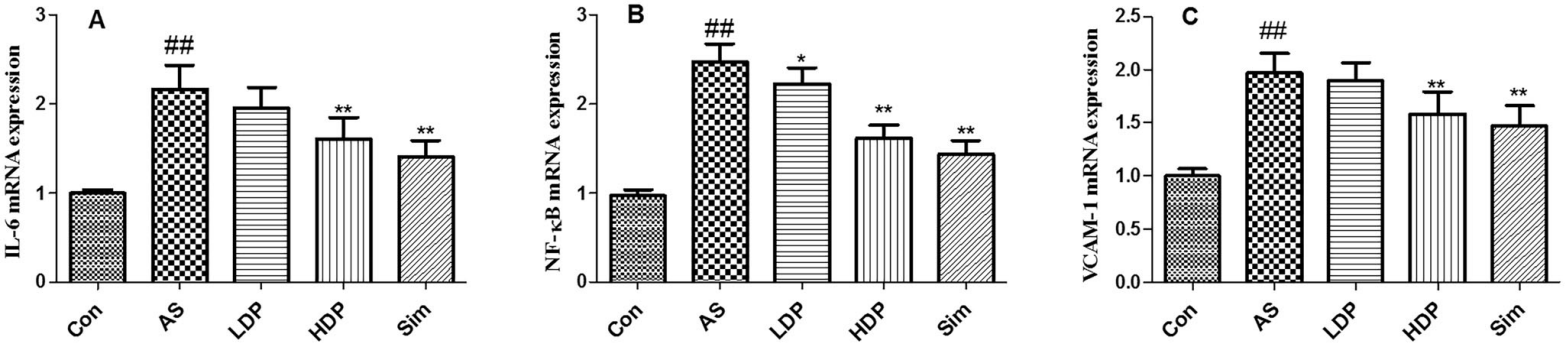
Delphinidin-3-*O*-glucoside (DP) down-regulated the mRNA expression of aortic IL-6 (A), NF-κB (B), and VCAM-1 (C). Values are expressed as mean ± *SD*. ^##^*p* < 0.01 *vs.* control (Con) group; *p < 0.05 *vs.* atherosclerosis (AS) group; **p < 0.01 *vs.* atherosclerosis (AS) group.

As shown in Figure 8, our results indicated that the mRNA expression levels of IL-6, NF-κB, and VCAM-1 were up-regulated obviously in AS group compared to those in the Con group (*p* < 0.01). However, administration of HDP or Sim down-regulated these aortic inflammation-relevant gene expression in HFD-fed rabbits (*p* < 0.01).

## Discussion

AS, a major cause of cardiovascular disease and remains one of the most complex diseases in the world (Karagkiozaki et al. 2015). Although the exact mechanism of the pathology of AS remains unclear. The increasing evidence has shown that lipid metabolism, oxidative stress, and inflammation were involved in the development of AS (Marchio et al. 2019). Therefore, the alleviation of hyperlipidaemia, inflammation, and oxidative stress has been proposed as an effective therapy for the management of AS disease. The present study used an HFD-induced rabbit model to investigate the prophylactic effect of DP on the development of AS. Our findings demonstrated that DP prevented atherogenesis, the inhibition of AS may be associated with the suppression of oxidative stress and inflammation.

DP is an anthocyanidin in the extract of *Hibiscus sabdariffa* calyces, which exerts multiple biological activities, including antioxidant and anti-inflammatory effects. Dyslipidemia is one of the primary risk factors for coronary AS. Hypertriglyceridaemia plays a vital role in the proatherogenic mechanism of AS. High blood lipid levels could evoke lipid deposition and endothelial injury of the vascular wall, eventually resulting in the formation of atherosclerotic plaques (Peng et al. 2017). Epidemiological studies demonstrated a positive correlation between the serum TC, LDL-C levels, and the severe degree of AS (Lu et al. 2018). Besides, previous studies have revealed that the decrease of serum lipid profiles could prolong the lifetime and inhibit the clinical complications of AS (Illingworth and Bacon 1987). In the present study, our results indicated that the DP decreased TG, TC, LDL-C, HDL-C, liver TG, TC, and aorta TG, TC. In addition, we also observed DP improved aortic lesions in HFD-fed rabbits. The hypolipidemic effect of DP was in agreement with a previous study, which revealed that DP improved lipid metabolism via the regulation of fatty acid oxidation genes *in vitro* (Harada et al. 2018). Therefore, our results indicated that DP improved atherosclerotic lesions in HFD-fed rabbits which may be caused by the hypolipidemic effect of DP. These findings showed that DP suppressed the development of AS via improving lipid metabolism *in vivo*.

Oxidative stress has long been considered an important etiology of AS, which plays a vital role in the development of AS (Kattoor et al. 2017). It has been demonstrated that oxidized lipids were involved in immune and inflammation response processes, and accelerated the pathological progression of AS (Hajjar and Gotto 2013). In fact, oxidation of LDL is one of the primary factors for the pathogenesis of AS (Steinberg 2009). Oxidized LDL (oxLDL) was considered the main atherogenic lipoprotein in the arterial wall (Hartley et al. 2019). Therefore, the suppression of oxidative stress might be an effective method to decrease lipid accumulation and ultimately alleviate the severity of AS. MDA was a lipid peroxidation product that was used as a marker of oxidative injury (Tsikas 2017). CAT, GSH-Px, and SOD were important antioxidant enzymes that protected cells against oxidative injury (Wu et al. 2018). In the present study, DP decreased the MDA content and enhanced the activities of CAT, GSH-Px, and SOD in HFD-fed rabbits. These results were supported by a previous study, which reported that DP alleviated oLDL-induced apoptosis and oxidative stress in vascular endothelial cells (Xie et al. 2012). Our findings showed that the antiatherogenic effect of DP may be associated with the suppression of oxidative stress.

AS, is a chronic vascular inflammatory disease, which is related to endothelial dysfunction. Previous reports have indicated that inflammation response was associated with the progression of AS (Raggi et al. 2018). The abnormal inflammatory response plays an important role in the initiation and development of AS (Haddy et al. 2003). Besides, pro-inflammatory mediators, such as TNF-α and IL-6 which accelerated the inflammatory response were also increased in AS patients (Spagnoli et al. 2007; Kals et al. 2008). CRP could evoke endothelial cells to generate chemokines and adhesion cytokines (Wang et al. 2019). It has been reported that endothelial cells activation also caused the secretion of leukocyte adhesion molecules, including ICAM-1 and VCAM-1. MCP-1 plays a vital role in atherosclerotic plaque formation. The levels of MCP-1 are increased after inflammatory response which is related to the atherosclerotic lesion (Lin et al. 2014). NF-κB has long been recognized as a pro-atherogenic factor and NF-κB activation could facilitate the generation of downstream inflammation-related cytokines, including MCP-1, VCAM-1, CRP, IL-1β, and TNF-α, resulting in the development of AS lesions (Yu et al. 2015). Besides, the previous study has indicated that the alleviation of inflammation could prevent the progression of AS *in vivo* (Paoletti et al. 2004). Therefore, the suppression of pro-inflammatory cytokines might be a novel intervention in the prevention and treatment of AS (Voloshyna et al. 2014). As reported in a previous study, delphinidin exerted beneficial effects on processes causing chronic inflammation in patients with cardiovascular risk factors (Dayoub et al. 2017). Our findings were in line with the previous study that showed that DP not only prevented the progression of AS but also inhibited inflammatory cytokines (ICAM-1, MCP-1, VCAM-1, CRP, IL-6, and TNF-α) and NF-κB activation in the aorta of HFD-fed rabbits, suggesting that the antiatherogenic effect of DP may be associated with the inhibition of the inflammatory response.

## Conclusions

The natural compound DP derived from *Hibiscus sabdariffa* calyces exerted a prophylactic effect in the atherosclerotic rabbits induced by HFD. Our results indicated that the anti-atherosclerotic effect of DP is partially related to the improvement of lipid metabolism, the alleviation of oxidative stress and inflammation. These findings implied DP may be considered as a novel agent for the clinical therapeutic strategy of atherosclerosis diseases treatment. Besides, this study also provided a theoretical basis for further development of DP as a novel anti-atherosclerosis drug.
